# Reply: Cancer care in rural areas

**DOI:** 10.1038/sj.bjc.6601770

**Published:** 2004-04-06

**Authors:** N C Campbell, P A Kiehlmann, L Stevenson

**Affiliations:** 1 Department of General Practice and Primary Care, University of Aberdeen, Foresterhill Health Centre, Westburn Road, Aberdeen AB25 2AY, UK; 2 Northeast Scotland Cancer Co-ordinating and Advisory Group (NESCAG), Cancer Network Office, Aberdeen Royal Infirmary, Foresterhill, Aberdeen, UK

Sir,

The experiences of Colomer *et al*, with a flexible combination of local and central delivery of chemotherapy agreed at a local level, are an encouraging example of a way forward in cancer treatment provision to rural areas. We reported many agreed priorities for cancer care in rural areas in our paper, of which agreed multidisciplinary protocols for chemotherapy in local areas was one (Stevenson *et al*, 2003). The abilities of Colomer *et al* to reach agreements between three centres and community hospitals are to be commended particularly in light of the polarised views that we reported (despite taking care not to exclude combinations of local and central delivery of chemotherapy in the wordings we used and by further qualitative analysis of opinions expressed during interviews). In Scotland, the divergent views of practitioners caring for patients with cancer are mirrored by those of patients (Bain *et al*, 2002), and appear to be responsible for substantial variations in provision even within rural locations (Smith and Campbell, 2004). This variation in practice stems from a real lack of evidence about the risks, benefits and costs of local chemotherapy provision, as well as uncertainty about how to balance uncommon but potentially serious risks against less easily measured effects on quality of life. As ever, where there is genuine uncertainty about the correct course of action, more research is needed. In the meantime, locally agreed arrangements, like those achieved by Colomer *et al*, could help to remove local inconsistencies, clarify protocols for rural practitioners, and reduce the uncertainties felt by many.

## References

[bib1] Bain NSC, Campbell NC, Ritchie LD, Cassidy J (2002) Striking the right balance in colorectal cancer care – a qualitative study of rural and urban patients. Fam Pract 19: 369–374 12110557 10.1093/fampra/19.4.369

[bib2] Smith SM, Campbell NC (2004) Provision of oncology services in remote and rural areas: a computer assisted telephone survey. Eur J Cancer Care, (in press) 10.1111/j.1365-2354.2003.00472.x 15115475

[bib3] Stevenson L, Campbell NC, Kiehlmann PA (2003) Providing cancer services to remote and rural areas: consensus study. Br J Cancer 89: 821–827 12942111 10.1038/sj.bjc.6601166 PMC2394474

